# Biological characteristics of IL-6 and related intestinal diseases

**DOI:** 10.7150/ijbs.51362

**Published:** 2021-01-01

**Authors:** Yuexin Guo, Boya Wang, Tiantian Wang, Lei Gao, Ze-jun Yang, Fei-fei Wang, Hong-wei Shang, Rongxuan Hua, Jing-dong Xu

**Affiliations:** 1 Department of Oral Medicine, Basic Medical College, Capital Medical University, Beijing 100069, China.; 2 Undergraduate Student of 2018 Eight Program of Clinical Medicine, Peking University Health Science Center, Beijing, 100081, China.; 3 Department of Physiology and Pathophysiology, Basic Medical College, Capital Medical University, Beijing 100069, China.; 4 Department of Bioinformatics, College of Bioengineering, Capital Medical University, Beijing 100069, China.; 5 Department of Clinical Medicine, Basic Medical College, Capital Medical University, Beijing 100069, China.; 6 Experimental Center for Morphological Research Platform, Capital Medical University, Beijing 100069, China.

**Keywords:** Interleukin-6(IL-6), Intestinal diseases, Inflammation, Tumor, Nerve Immune barrier

## Abstract

The intestine serves as an important digestive and the largest immune organ in the body. Interleukin-6(IL-6), an important mediator of various pathways, participates in the interactions between different kinds of cells and closely correlates with intestinal physiological and pathological condition. In this review we summarize the signaling pathways of IL-6 and its functions in maintaining intestinal homeostasis. We also explored its relation with nervous system and highlight its potential role in Parkinson's disease. Based on its specialty of the double-side influences on intestinal tumors and inflammation, we summarize how they are done through distinctive process.

## Introduction

Nearly all of the nutrient digestion and absorption are carried out in the intestine tract and abundant microbes parasitic in the gastrointestinal (GI) tract may be involved in the process. Through this complex process microbial metabolites, together with remains in the digestive tract, interact with host cells widely and even a seemingly small dysregulation may lead to the breakdown of intestinal homeostasis just like the 'butterfly effect' 1. The maintenance of this precise balance requires the control of epithelial cells via different immune mechanisms constituting the intestinal immune barrier. Though there is no common definition of the intestine immune barrier, here we put its contents as the mucous layer, immune cells and tissues and immunoactive substance 2. Immune cells are further distinguished different immune cell types or cell type-specific subsets based on the expression of cell surface and intracellular markers. With a wide variety of biological functions, IL-6 is the hub in mediating the communications among these cells and the cross-talk between intestinal epithelium and the gut microbiota. Not limited to the gastrointestinal tract, its receptor, IL-6R, is widely expressed, which causes inflammation in response to a wide variety of stimuli including infection, stress and trauma 3, for example, in the liver leading to the acute phase protein response, in the hypothalamus together with IL-1β leading to systemic fever 4, and in the gut leading to Th17 activation 5. Within the scope of this review, we just limit it to the functions related to the intestine.

As a polypeptide, IL-6 consists of α and β chains whose structure has been fully elucidated and used for targeted therapy widely 6, 7. It can be secreted by several kinds of cells, from intestinal epithelial cells to lymphocytes, and a large amount of studies has offered promising targets. However, as it is difficult to control the multiple sources, there is still a long way to go from theoretical feasibility of pharmacology to clinical application. In this review, we focused on the role of IL-6 in the function and overlapping relation with intestinal dysfunction.

## Discovery and structure of IL-6

When Toshio Hirano* et al*. first reported the molecular cloning, structural analysis and functional expression of the cDNA encoding B-cell stimulatory factor 2(BSF-2), they deduced that it is a novel interleukin containing 184 amino acids 8. At that time, the dideoxy method and gas-phase protein sequencer were used to obtain the complete neucleotide sequence of the insert cDNA as well as the partial amino-acid sequence of each fragment of the BSF-2. With those methods researchers further compared the protein sequence of BSF-2 with all proteins in the National Biomedical Research Foundation Database (NBRF) and Genetic Sequence Data Bank (GSDB) but no strong sequence similarity was revealed in the comparison. Then its many other functions such as promoting the proliferation of hepatocytes were verified and they are not limited to plasma cells. As most of them are related to the acute phase protein and interactions among lymphocytes, it was finally termed as lnterleukin-6 (IL-6). With technology advancing, its structure has been fully illuminated 6 and the history of its discovery has also been specially reviewed 9, which may have strong implications for future investigations.

## Maintaining homeostasis in physiological state (Function of IL-6)

In normal conditions, serum IL-6 concentration is about 1.6pg/ml, narrowly mediating mild immune responses in defense of invasive pathogens 10. Based on its interaction with vagus 11, it may also impacts the smooth muscle cells or secretory cells and then consequently the intestinal motility or secretion.

### Participating in the immune-epithelial-bacteria function regulation cross-talk

Gastrointestinal tract, an essential part of digestive system, is mainly responsible for absorbing nutrients. A total number of about 10^13^-10^14^ CFU/mL bacteria residing in the intestinal tract are involved in this process, whose metabolites together with food residues make up most of contents in the intestine directly or indirectly in contact with the mucus layer isolated from epithelial cells. IL-6 plays an essential role during the immune-epithelial-bacteria cross-talk in both the beneficial and harmful processes carried out by different types of microbiota. Pediococcus acidilactici K15, selected as the most effective strain of lactic acid bacteria for stimulating IgA production, induce the secretion of IL-6 in BDCA1^+^ DCs (mDC1s) via its double-stranded RNA and enhance the production of protective IgA by B plasma cells 12. As excessive IL-6 may initiate inflammation, another experiment by Vinderola et al. showed increasing level of iIgA-producing cells without corresponding enhancement in the CD4^+^ T-cell population in mice after orally administered strains of lactobacillus (LAB), indicating that the effect was limited to plasma cells secreting IgA. Further comparation between the influence by LAB and E.coli found that higher level of IL-6 secreted in a TLR2-dependent manner is responsible for the enhancement of IgA and this could also be seen in other probiotics such as L. casei CRL 431 and L. helveticus R389 13. IL-6 level induced by these probiotics variety is precisely enough for B cell differentiation but not adequate for T cells, consequently maintaining the immune response to a proper extent. However, with IL-6 being able to promote inflammation progress most of the time; many probiotics alleviate intestinal pathology via reducing its level. Forit was verified that the mixture of Lactobacillus mucosae NK41 and Bifidobacterium longum NK46 from human feces significantly mitigated IL-6 level and immobilization stress (IS)-induced anxiety-like/depressive behaviors 14. And short chain fatty acids (SCFAs) generated by bacteria such as L. acidophilus KLDS1.0738 15 act as stimuli for macrophage and reduce IL-6 secretion *in vivo* which is studied using mice. In turn, injection SCFAs has achieved promising results that allow for further clinical trials 16. Despite those beneficial microflora, some metabolisms or its own metabolisms serve as pathogens, cause inflammation and put the intestine in danger, many of which involve the increase of IL-6 level 13, 17, 18, But exceptions also exist, such as that in Lycium barbarum polysaccharides (LBPS) treatment of cyclophosphamide (CTX)-induced mice, the increasing level of IL-6 is concomitant with abundances of Bacteroidaceae, Lactobacillaceae, Prevotellaceae and Verrucomicrobiaceae positively associated with immune traits 19.

The imbalance of those beneficial and harmful species is regarded as the origin for many diseases and clinical trials found mitigatory symptoms in various intestinal diseases such as UC and colorectal cancer after probiotic treatment, always accompanied by lower IL-6 level 20, 21. But this kind of treatment remains conflicting, suggesting more multifaceted interactions between bacteria and intestinal immune system in which IL-6 serve as an important mediator 22. Further, much detailed work remains to be done due to unique characteristics of microflora as even the same bacteria family can exert opposite influence on the same cells for its complex compositions and interactions, such that in the Bifidobacterium adolescentis strain IF1-11 induced significantly higher IL-6 and lower IL-10, contrary to that in IF1-03 23.

Also considering that the bacteria-associated treatment mainly modulates the long-term intestinal functions while the drugs targeted IL-6 are generally used to alter the acute symptoms, combined treatment of them may offer the patients better therapeutic efficacy.

### Maintaining mucosal integrity

The mucous layer serves as the out-most colonic barrier exposed to pathogens and contains mainly mucin2 (MUC2) secreted largely by the goblet cells with toll-like receptors (TLRs). Studies have demonstrated that the IL-6 mediated Jak/STAT3 pathway may drive goblet cells differentiation via its downstream PI3-kinase/Akt signal peptide corroborating the previous finding of visible damage of mucosa in IL-6^-/-^ mice 24. The second defensive barrier are the junctions between epithelial cells including tight junction (TJ), adheion junction, desmosomes connection and gap junction from top to basement. TJ, deemed to be cardinal, is composed of occludin, claudins, junctional adhesion molecule (JAM), zonula occludens (ZO) and limits the passage of macromolecules 25 and microorganism 26, 27. TJs are important intestinal barrier against exogenous pathogens and largely determine the intestinal permeability modulated by IL-6. To investigate the underlying mechanism between them, researchers carried out experiments both *in vivo* and *in vitro* (consisting of filter-grown Caco-2 monolayer), corroborating that IL-6 enhance the intestinal TJ barrier in a size dependent manner relying on the increasing expression of claudin-2. IL-6 caused about a 3- to 4-fold increase in trans-epithelial flux of smaller-sized paracellular marker urea without affecting the flux of larger-sized molecules including mannitol, coinciding with the factor that claudin-2 dependent pore pathways allowing paracellular flux of ions and smaller sized molecules <4.0 Å in molecular radius also indicating that the increasing permeability is limited to smaller-sized molecules. They further verified that IL-6 activated JNK (c-Jun N-terminal kinase) signaling pathway, leading to the activation of transcription factor AP-1 and consequently a sequential activation of claudin-2 gene. Despite the same finding that IL-6 increase TJ permeability via enhancing claudin-2, Takuya Suzuki *et al.* attested the up-grading level of claudin-2 gene in a MEK/ERK and PI3K-dependent manner with the involvement of transcriptional factor Cdx2 (caudal-related homeobox-2) 28. Considering that Cdx2 is only expressed in the epithelial cells at the luminal surface in normal colons but is found in the colonic crypt in patients with Crohn disease (CD) and ulcerative colitis (UC), and that its expression in mice intestine also correlates with claudin-22, the different models they used may account for the distinctive outcomes in two studies 29. In fact, besides the role in physiological conditions, IL-6 also pathologically promotes the mucosa preservation and facilitates mucosal repairing, which will be discussed in part 3.3. Composing both phospholipid (PL) and protein, integrity of membrane in intestinal epithelial cells can also be influenced by IL-6 mediated alteration of secretory phospholipase A2 (sPLA2). Researchers believed that IL-6 leads to changing in the composition of (PL), more exactly, an increase in phosphatidylethanolamine (PE) and sphingomyelin (SM) and a decrease in phosphatidylcholine (PC) and lysophosphatidylcholine (LPC) 30. PE as well as SM serves as inflammatory signaling factors in IBD suggesting that they may function as mediators of the ascending level of sPLA2 induced by IL-6. Previous studies also found this enhancement in IL-6 level was achieved via the regulation of C/EBP-β dependent on epithelial detachment rather than certain proteases 31.

Apart from the induction of proteins as junctions, IL-6 up-regulate the expression of several genes responsible for proliferation and anti-apoptosis after treatment with C.* rodentium*, including the Bcl family members Bcl-xL and Mcl-1 (but not Bcl-2), the IAP family member cIAP-2 (but not survivin), and the NF-κB family member, Bcl-3 32. Also a meta-analysis showed no difference in IL-6 level whether in the appearance of probiotics or not in the colorectal cancer after operation, so considering the various types of probiotics more human experiments with the control of single strain are demanded 33.

Despite the fact that most pathogens are resisted by integrated mucous layer, the remaining ones still cause danger to epithelium and their removal require the participation of other epithelial cells by direct phagocytose or initiating immune responses. During the process, IL-6, an indispensable important factor, involves in most of the immune response and a heavy load of researches have given detailed descriptions 34. In fact, apart from the well-known role in inflammation and tumor which we will discuss in part 3 and 4, IL-6 is required for tissue repairing after injury for its induction of intestinal epithelial proliferation. Here we just highlight its relationship with Paneth cells, the ones exclusively exist in the intestine crypt 35. Recently Jeffery* et al.* verified its role in the maintenance of the crypt stem cell niche using mice *in vitro* crypt organoid and *in vivo* models 36, 37 This is in the control of autocrine IL-6 secretion through the Wnt signaling pathway, supporting previous hypothesis that besides the role in promoting intestinal regeneration after injury, IL-6 also play a part in modulating crypt homeostasis 38. To determine the impact of IL-6R signaling on intestinal proliferation and repair under exogenous stress, it was exposed IL-6R^fl^ and IL-6R*^ΔIEC^* mice to DSS, the results indicated that the mice of both genotypes started to lose weight, but IL-6R*^ΔIEC^* mice seemed to cope better with DSS-induced colitis than WT as they significantly lost less weight than IL-6R^fl^ mice.

Originally termed as B-cell stimulating factor-2 (BSF-2), IL-6 first came to be known as irritation for B cells to secrete immunoglobulin (Ig) 39. Then it gradually won the name of cytotoxic T-cell differentiation factor for its role in inducing T cells maturation 40. Now IgA, M, D, E and G have been recognized for their roles in polymerizing and adhering pathogens though alterations of different kinds of Ig varied among experiments in UC 41. Also IL-6 is generally acknowledged to be the transmitting factor which induces the proliferation and differentiation of plasma cells from immune cells like macrophages 42. Most of the subsets and their respective functions have been clarified, and what is deserved to be mention is a recent investigation on the connection among IL-6, T helper17 cells (Th17) and T regular cells (Tregs) for Th17's unique characteristic as secreting IL-17. The break of this precise balance has a close correlation with inflammation and we will discuss it in part 3.2.

**Figure 1:**
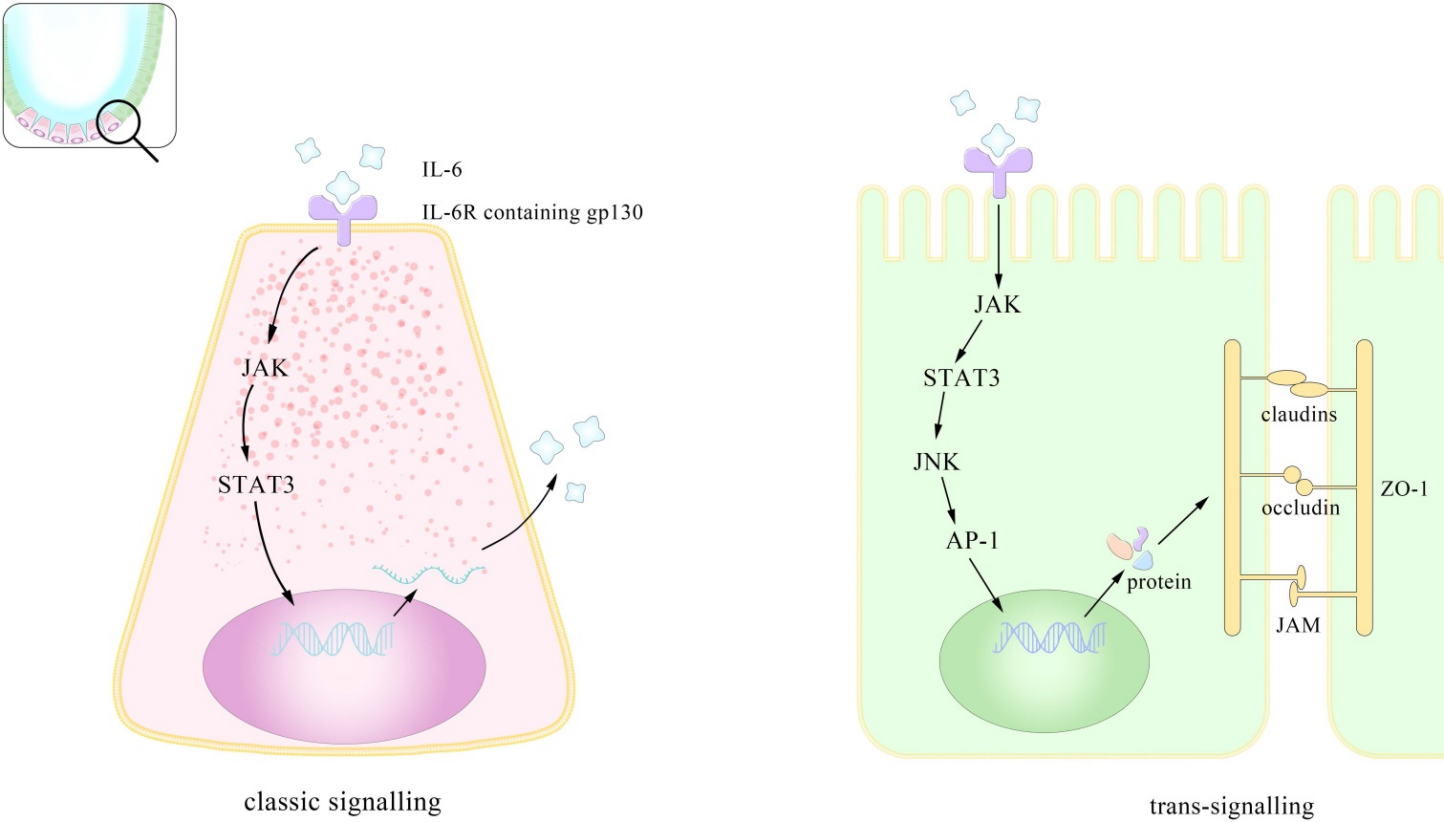
** Pattern of mechanisms of IL-6 promoting the integrity of the intestinal epithelium.** IL-6 assists maintain intestinal homeostasis through both classical and cross-signaling pathway. This is the enlarged view of the role IL-6 plays in the intestinal epithelium. IL-6 promotes the proliferation of paneth cells in the crypt through (JAK/STAT3 classic signaling). This preserves the intestinal integrity by inducing gene transcription via trans-signaling (sIL-6R: soluble IL-6 receptor; ZO: zonula occludins).

Based on its close connections to multiple cells and functions, IL-6 related targeted treatments have received long-term attention. However, several trails have shown that its blockade may lead to unveiled by-effects unless more specific antibodies or drugs are found or synthesized. In brief, Figure 1 showed that IL-6 help, with the maintenance of intestinal homeostasis through both classic signaling and trans-signaling pathways.

### Receptor and signaling pathways of IL-6

Researchers have corroborated IL-6 signaling in three pathways, classic-signaling, trans-signaling and trans-presentation. All of the three ways above demand the combination of IL-6, monopeptide glycoprotein IL-6 receptor (IL-6R), and glycoprotein130 (gp130) (mainly site III) and the differences lie in the existing forms of the substances as well as their differentiated functions as a result. The classic signaling requires membrane IL-6R and is normally seen in leukocytes and liver cells while the trans signaling utilizes soluble IL-6R (sIL-6R) and plays important roles in times of tumors. The dose response curve for the classic one is bell-shaped coinciding with the fact that high concentration of IL-6 induces the cleavage of sIL-6R and consequently the trans-signaling pathway 43. Both the two ways involve the formation of a complex hexameric formed by double sequentially combination of IL-6, IL-6R and gp130 and further activate different downstream pathways related to gene transcription 44. The downstream pathways include JAK/signal transducer and activator of transcription (STAT3), Src homology 2 domain-containing protein tyrosine phosphatase-2 (SHP-2)/extracellular-signal-regulated kinase (ERK) mitogen-activated protein kinase (MAPK), phosphoinositol-3 kinase (PI3K)/protein kinase B (PkB)/Akt and SRC/YAP 45, 46. In JAK/STAT pathway the major activation is performed on STAT3 and one of its downstream molecules, the suppressor of cytokine signaling (SOCS3), can inhibits the catalytic activity of JAK. Controlled by gene DNMT1 47, the expression of SOCS3 serves as a natural inhibitor of STAT3, with the same effect happening in the protein inhibitors of activated STAT (PIAS) 24. Intriguingly a clinical investigation showed that SOCS3 level increased in UC but dropped in UC-CRC. SOCS3 gene methylation may account for its decrease in UC-CRC and its increase in UC likely explains the lesions but this contrary alteration overall suggests a more complex association between IL-6 and SOCS3 48. Besides SOCS3, there exists another kind of natural inhibitor of IL-6 signaling. By binding to the complex of IL-6/sIL-6R, it selectively block the trans-signaling with high affinity and studies have shown its concentration highly related to the tumor progress 49. Although recombinant sgp130 has been used in the treatment of inflammation, illuminating the modulating factors of it *in vivo* may benefit the immunotherapy against tumor 50.

**Figure 2:**
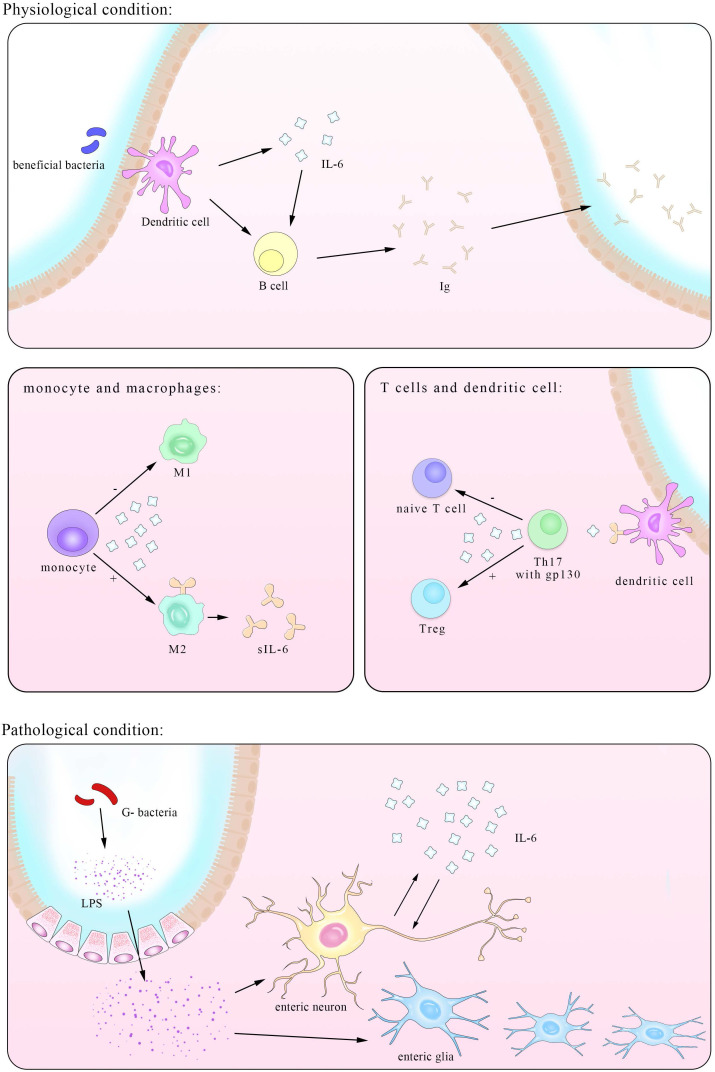
** An illustration of the IL-6-involved in cross-talk between intestinal bacteria and immune cells in both physiological and pathological conditions.** IL-6 has close interactions with ENS within both enteric neurons and enteric nervous glia. The trans-presentation signaling pathway between DCs and naïve T cells is also showed here, suggesting a pivotal role of T cells in IL-6 mediated inflammatory process.

The third pathway recognized recently involves similar molecules but in different cells. As figure 2 shown that A subset of CD11b^+^ DCs that are Sirpα^+^ express IL-6Rα which bound to IL-6 and then presented to the gp130 on myelin peptide specific encephalitogenic T cells. Although classic IL-6 signaling is sufficient to suppress the transforming growth factor β (TGF-β)-induced expression of Foxp3, this process is proved indispensable for the priming of pathogenic T_H_17 cells 51.

Many blockers in this process have been synthesized or discovered and experiments aimed at testing their efficacy and side effects are also operated on a large scale. Not only the antibody targeted directly on IL-6 but also the one on its membrane receptors and the components of its upstream activators, such as JNK, extracellular signal-regulated kinases (ERK) and p38 are intensively researched. Although blockers of critical molecules in the downstream pathways mentioned above are meticulously studied with reviews for comprehensive summary 6. Now several approaches such as genetic knockout and antibody incubation have been put into use clinically and could probably offer patients a bright prospect.

## Relevant to inflammation associated diseases

The gut wall is protected by a well-developed immune system and an intestinal mucosal barrier composed of epithelial cells and mucus layers secreted by goblet cells, and this contains commensal bacteria that regulate the passage of fluids, macromolecules and antigens. It may help in limiting bacterial colonization by releasing mucus, antimicrobial peptides and immunoglobulins 52. Normally it assists with the elimination of pathogens but long-term inflammation such as the CD and UC may lead to a serious of pathological manifestation seriously affect the patients quality of life. IL-6 is normally known to aggravate inflammation by both directly driving lymphocytes proliferation as well as differentiation and directly/indirectly through nervous system. Nevertheless, the anti-inflammatory functions like fresh for many people, was gradually unveiled. Like a key hub in the busy transporting net, IL-6 acts as a mediate in many functions in the gastrointestinal immune barrier 53-55.

### IL-6 as a vital mediator between the neuro-immune axis

Apart from the well-known regulatory role in immune system, IL-6 is an important mediator in the interactions between nervous system and immune system in both direct and indirect ways. Overwhelming studies have demonstrated highly bi-directional relationship between intestinal diseases and mental diseases accompanied with neuron lesions 56. It is reported that increasing IL-6 level in adult mice intestine is secreted by enteric glia with the stimuli of IL-1β or LPS 57, 58. Intriguingly, exogenous IL-6 suppresses the expression of IL-6 mRNA in a dose-dependent way. Another experiment by Burgueño *et al.* further demonstrated that upon treated with LPS, enteric neurons expressing TLR2/4/9 secrete IL-6 in NF-kB dependent pathway. But the way IL-6 secretion is not limited to this for pretreated with Bay 11-7082 largely reduced the production of IL-6 but not completely abrogated it 58. Besides the direct secretion of IL-6, nerves inhibit IL-6 expression in lymphocytes, based on the fact that Ach receptors are found on them and that IL-6 level in vagotomized mice were higher than that in sham-operated mice 59. Activation of β-adrenoceptors protects the intestine from the detrimental effects by inhibition of IL-6 elevation as well as other cytokines 60 and acetylcholine is found to have the same impact in a post-transcription manner depending on MФ nicotinic acetylcholine receptor (alpha7nAChR) 61.

Besides the impacts ENS exert on IL-6 secretion, IL-6 in turn affects ENS via several ways. In dissected and dissociated myenteric plexus of newborn rats, the highest average neurite outgrowth per neuron was observed in GDNF-treated and Hyper-IL-6-treated cultures, indicating an essential role it plays in ENS survival and differentiation 62. And IL-6 is capable of reducing the expression of vasoactive intestinal peptide (VIP) in biopsies of CD patients and healthy subjects, although the precise source remains unconfirmed 63. VIP is an important peptide in the brain-gut axis and its function in repressing the activation of tumor-associated macrophages is also reported 64. IL-6 can also exert direct excitatory effect on a subset of myenteric neurons and reversibly promote the presynaptic inhibition of acetylcholine released from cholinergic nerve terminals 65. It also suppresses norepinephrine release by myenteric nerves in a dose-dependent way and with IL-1β synergizing, subthreshold level of IL-6 can also exert similar influences as in higher concentration 66. Taken together, these findings suggest that IL-6 is an important neuromodulator of gastrointestinal motility which correlates closely with many diseases within and outside the gastrointestinal tract (refer to Box2 for more details).

Moreover, higher level of IL-6 is witnessed in irritable bowel syndrome (IBS) patients but its correlation with gastrointestinal dysfunction remains unknown. Researchers using maternal separation (MS) mice found increase activity of submucosal neurons is partly dependent on IL-6 in a Ca^2+^-mediated manner. This process requires membrane-bound IL-6R which is expressed predominantly in neuronal fibers and further initiates different pathway for gene transcription of various functions 67, 68. Noticing that the calcium response could be potentiated by the corticotropin-releasing factor (CRF) 69, further studies investigated the cross-talk between IL-6 and CRF in colonic submucosa neurons and found that IL-6 also potentiate CRF-induced calcium response and CRF stimulates the colonic secretion of IL-6 by submucosal neurons and T-helper lymphocytes with CRFR1. It also enhances IL-6R on neurons and activates IL-6 induced MAPK but not STAT3 pathway 70. Studies using LPS or directly administration of IL-6 showed its role in hypothalamic-pituitary-adrenal axis(HPA) activation but as those IL-6 could hardly been restricted to intestine, we will not give more descriptions of it due to the scope of this review 71. Similar studies have convinced more and more places where nerve physiological conditions are related to IL-6 levels, but unfortunately many cytokine concentration alterations emerge simultaneously so it is hard to make variety control 63. The use of monoclonal antibodies or gene knockout technology is likely to solve the problem. Also now a large proportion of articles concerning the impact of IL-6 on neurons are confined to the CNS, so more attention to peripheral nerves probably shed light on novel therapies.

#### Box 2: IL-6 and gastrointestinal motility

Gastrointestinal motility is essential for food digestion and daily detoxification and its stasis is frequently concomitant with multiple organ dysfunction syndrome (MODS), which show marked up-regulation of IL-6 correlated with reduced colonic motility and probably result in gastrointestinal smooth muscle dysfunction 72. Although IL-6 has no impact on the spontaneous contraction of colon in normal rats, it has conflicting influences in different disease models 73. Studies showed that higher IL-6 level could be induced by damaged muscle fibers and neutralizing IL-6 receptor antibodies (xIL-6R) improved the symptoms in Dystrophin-deficient C57BL/10ScSn-Dmd^mdx^/J (mdx, dystrophic) mice 74. Another type of intestinal dyskinesia is postoperative ileus (POI) accompanied with higher IL-6 level, and IL-6 mRNA from muscularis extracts demonstrated a significant induction after intestinal manipulation 75, 76. The negative correlation between IL-6 level and muscle contractility was also verified in an experiment using repeated LPS injection to induce muscularis cross-tolerance to POI. And this relation, mainly via the NF-kB, was not shown in mucosa, indicating the exclusive effects IL-6 exert on the muscularis 77, this phenomenonis also elucidated in UC 78 as well as diseases in other organs^79^ and various kinds of colonic damages 80, 81.

Many systemic diseases also show alterations in gut motility. Colonic dysmotility accompanied with significantly elevated blood plasma IL-6 levels occurs in type 1 diabetes and studies demonstrated the increased contraction of distal colon mediated by IL-6 in the process 73. Similar to IBS, exogenous IL-6 in myenteric plexus also facilitates contraction of proximal colon in chronic unpredictable mild stress (CUMS) induced depression model, coinciding with the higher level of IL-6 in depressed patients 82. Considering the Ca^2+^ response IL-6 could induce and findings that increased Ca^2+^ by ERK and p38MAPK pathways lead to colonic smooth muscle hypercontractility, it may be reasonable provide an explanation and even a treatment for those findings mentioned above 83.

### IL-6 involved in lymphocytes orchestration and promoting proliferation or differentiation

Inflammatory bowel diseases (IBD), including UC and CD, a kind of chronic disease in intestine, seriously diminish patient's life quality. They are tricky clinical problems due to ambiguous causes and lack in radical cure, indicating a complex etiology lying behind. Multiple factors such as susceptible genes 84, 85, dietary habits and psychological condition 86, 87 are shown to be relative although clear pathology remains unveiled. Both CD and UC share common symptoms like recurrent abdominal pain and constipation or diarrhea in the intestine accompanied with multiple uncertain systemic diseases that universally occurred in gastrointestinal dysfunction. However, they also distinguish from each other in many aspects, such as typically CD is profiled by more T helper1 (Th1) and Th17 compare with Th2 whereas UC is characterized by a majority of Th17 and Th2. The up-regulated level of IL-6 has been confirmed in both diseases and depletion of IL-6 alleviates both symptoms, suggesting a pivotal role it plays in the process 7. Studies confirmed that macrophages (Mφ) and CD4^+^ T cells are major sources of IL-6 in IBD, with Mφ being mainly influenced by macrophage-migration inhibitory factor (MIF). IL-6 polarized Mφ into a pro-inflammatory type M1 which secret cytokines and molecules aggravating inflammation 88. In fact, with the expression of mIL-6R decreasing in epithelial cells in chronic colitis (CC) and colitis-associated premalignant cancer (CApC) mice model, sIL-6R shed mainly from the Mφ are important for the downstream effects of IL-6 89. These include the enhanced expression of anti-apoptotic genes *Bcl-2* and *Bcl-xl* in T cells via STAT3 pathway while *STAT3*- independent pro-apoptotic gene *Bax* was unaffected 90. IL-6 also involve in the disease progress by regulating T cell differentiation, for it is demonstrated that IL-6 in combination with TGF-β triggers STAT3 pathway and induces retinoid-related orphan receptor γt (RORγt) and RORa expression in Th17, a decisive transcription factor controlling the differentiation of Th17 lineage 91, 92, which may secrete pro-inflammatory cytokines including IL-17 and IL-6 thus increase the inflammatory response. Intriguingly, in IL-6^-/-^ mice, TGF-β sorely promote the generation of Foxp3^+^ and initiate Treg suppressing inflammation 5. In fact, further studies confirmed the role of IL-6 largely caused by disturbing the intercellular FOXP3 and chromatin-modifying enzyme EZH2 interaction 93, and CD103^+^CD11b^+^ DC serve as the main IL-6 source 94. Based on this, PF-04236921, a human monoclonal antibody against IL-6, was exploited to cure IBD in phase II RCT. Crohn's Disease Activity Index (CDAI)-70 response rates with PF-04236921 50 mg were notably improved than placebo despite later trails were forced to an end due to unreasonable abscess and perforation 95.

Besides the most typical T cells involved in IBD, IL-6 exert influence on many other cells. In CD, the secretion of IL-6 in epithelial cells leads to the reduction of intercellular cell adhesion molecule-1 (ICAM1) via NF-κB 96, 97. Also incubation with IL-6 result in lower expression of endothelial protein C receptor (EPCR) and thrombomodulin (TM) by the vascular endothelial cell (VEC) which consequently enhances coagulation and limit the anti-inflammation effect 41. These results hinted that treatment targeted IL-6 may bring patients a promising prospect.

Intestinal epithelial cells as well as immune cells like antigen presenting cells (for example monocytes 98 and Mφ 99) are major sources of IL-6 whose secreting mechanisms have been widely reported (refer to Box3 for more interactions between IL-6 and immune cells in the intestine). But studies in mice found that other types of cells including intestinal smooth muscle cells could also secrete IL-6 with the stimuli of IL-1β 100. This is conflicting since in another experiment, after infected with *Trichinella spiralis*, mice showed that no significant distinction between the euthymic and athymic despite the general enhanced expression level of IL-6 in the longitudinal muscle myenteric plexus (LMMP), suggesting a less essential role for its contribution in muscle growth and contraction 101.

#### Box 3: Interactions between IL-6 and intestinal immune cells in other diseases

Pathologically intestinal manifestations are related to various organ diseases. Immunohistochemistry in decompensated cirrhosis patients showed colocalization of IL-6 with CD68^+^ iNOS^+^ Mφ and IL-6 was predominantly present in CD11c^-^ cells 99. Another study in mice showed IL-6 specifically derived from DCs are crucial in defense of *Giardia duodenalis.* In mice infected with *Giardia Lamblia*, IL-6 generated by mast cells were necessary for intestinal protective immunity 102. In cirrhotic patients with portal hypertension (PHT), hepatic venous pressure gradient (HVPG) significantly correlates with abnormal gut permeability and higher IL-6 levels 103. And IL-6 contributed to colitis-induced platelet responses including thrombocytosis and platelet hyperreactivity via the maturation and activation of megakaryocytosis 104. Further studies demonstrated that IL-6, secreted by bone marrow-derived blood cells, mainly leads to accelerated thrombus development within the inflamed colon 105.

And the role of IL-6 is not limited to the digestive tract but in correlation with many diseases in other systems as well as some systemic diseases 106. For example, obesity induced IL-6 promote the polarization of Mφ into a tumor-promoting type and thus exacerbate CAC progression 107. In brief, altering the intestinal microenvironment are closely related to body physiological functions and IL-6 is an important mediator in both interior intestine and interactions between intestine and other organs just like heart and kidney.

### IL-6 as an important member of the anti-inflammatory effect

As mentioned above, apart from its physiological functions, the impact of IL-6 in mucosal repairing after injury are vital for body recovery and are an essential part of its anti-inflammatory roles. It is reported that probiotics like *Bacteroidales* can recruit IL-6 and enlighten TJ by promoting the secretion of claudin-1 and mucin-2 which consequently alleviate inflammation. This further convinced their former observation that the colitis mice with anti-IL-6 mAb treatment beginning at the onset of co-housing led to significantly greater weight loss and decrease in crypt number over time compared with control group 108. Diseases in other organs can also influence the intestinal epithelial permeability during which IL-6 possess an indispensable role. During hemorrhagic shock and resuscitation (HS/R), IL-6^-/-^ mice showed markedly attenuated tissue injury and intestinal permeability compared with the wild type (WT) group 109. However, there lies some difference in the inflammatory state among outcomes from various laboratories. Inflammation was down-regulated in IL-6^-/-^ mice after HS/R in the experiment by Yang *et al*., while this condition appeared after intravenous administration of IL-6 by Meng *et al.* 110. Considering the pleiotropical nature of IL-6, this distinction may arise from the dose of IL-6 they used.

Studies have found that exogenous IL-6 inhibit acute inflammatory responses and prevents ischemia/reperfusion (I/R) injury after intestinal transplantation. Although no differences are observed in TJ, the higher level of SOCS3 compared with control group and related lower serum level of pro-inflammatory cytokines may be a reasonable explanation 111. Other protective roles of IL-6 in I/R lies in its restoration of microvascular by oral administration and reduced apoptosis via increased *bcl* gene expression 112, 113. Also in systemic bacteraemia following haemorrhagic shock oral administration IL-6 alleviate the symptom by reducing bacteria translocation from the gut and with less bacteriological cultures within the mesenteric lymph nodes 114, 115.

In active inflammation, adenosine released in the intestinal lumen could induce IL-6 secreted by intestinal epithelial cells, which activated neutrophils degranulation by an intracellular Ca^2+^ flux 116. This recruiting and activation of neutrophil could exert double effects for both recovering after colitis in bacteria-depleted mice and secreting substances such as IL-6 stimulated by IL-1 17, 117. The relationship and underlying mechanisms between IL-6 and inflammation have received much attention and contrary opinions existed concerning whether classic-signaling promotes anti-inflammatory effects and epithelium proliferation 7, 118. Apart from the need for clarification on mechanisms, propagating drugs into clinical trials and applications is also a pressing work that cannot be neglected.

The controversy over the essential characteristics of IL-6 has received long-time attention. Here we summarize its roles in inflammation in Figure 2 and find that many of them are in fact precise balance.

## Double-edged sword effect of IL-6 in tumorigenesis

### Tumor-promoting effects

Epidemiological statistical results showed that patients with IBD have a higher risk of colorectal cancer (CRC), suggesting a close relationship between them 119. In fact, major interactions between lymphocytes mediated by IL-6 show paramount resemblance and the distinction mainly lies in the immunosuppressive influence IL-6 exerts on Mφ and T cells in times of colorectal cancer which promote the immune evasion of tumors. It is reported that IL-6 secreted by colorectal cancer cells enhance the phagocytic capacity and migration of Mφ using a monocyte-macrophage THP-1 cell model and human peripheral monocytes 120. These Mφ, derived from monocytes recruited also by IL-6, are actually M2-like tumor-associated macrophages (TAM) which could express a series of cytokines including IL-6 to promote tumor progression 121. Another experiment demonstrated that IL-6 synergize with TGFβ1 induced the generation of CD8^+^CD25^+^Foxp3^+^ T cells (T8reg) *ex vivo*. With an ability to suppress CD4^+^ CD25^-^ T cell proliferation and Th1 cytokine production, this kind of T8reg has strongly suppresses the function of immune system 122. Studies also found the number of CD8^+^T cells show marked decrease in IL-6 overexpressed tumors due to impaired infiltration induced by IL-6 123, consistent with the fact that this effect is significantly improved after STT, which will be depicted in details in Part 4.2.

Working mainly via the trans-signaling pathway in the regulation of TGF-β 124, IL-6/JAK/STAT3 signaling axis is at the core of regulating many gene transcription and expression that play crucial roles in the generation and development of tumor 125. And this axis exerts both inter-cellular and extra-cellular pro-tumor effects, coinciding with the results of previous clinical cases that increasing level of IL-6 in CRC patients indicates high mortality and bad prognosis 126, 127.

Various kinds of gene mutation are the radical reason of tumor generation and IL-6/JAK/STAT3 aggravate the influence by promoting excessive gene expression. Inter-cellular pro-tumor activities of IL-6 mainly concern mutant genes in charge of cell cycle, apoptosis and those proto-oncogenes and anti-oncogenes. There is a previous review mainly focusing on the role of STAT3 pathway 128, so here we just introduce p53 gene, the most frequently altered one in colorectal carcinomas, protect cells from canceration by inducing apoptosis and suppressing mitosis. However, in the presence of IL-6, p53 DNA exhibit methyltransferase and lose its viability, resulting in unlimited cell proliferation 129. The activation of genetic transcription in many cells via both autocrine and paracrine like a chain action also leads to the deterioration of diseases, like that happens in the truncated Retinoid X receptor-α (tRXRα) murine which show an up-level in IL-6 concentration through NF-κB pathway. This enhancement was also found in UC patients, indicating an increasing susceptibility of colorectal cancer for them 130. Apart from the inter-cellular impact, IL-6 promotes tumor invasion, agiogenesis and metastasis by enhancing the expression of a disintegrin metalloprotease 17 (ADAM17), vascular endothelial growth factor (VEGF) and matrix metalloproteinase (MMP). ADAM17 promotes the cleavage of IL-6R and thus enlarges the trans-signaling effects IL-6 exert 131. Also considering previous reports that IL-6 potentiated the cross-talk between Mφ and tumors in other kinds of cancers, it is reasonable to inquire whether these happened in IBD and CRC 132. Further investigations in answering these questions will benefit the clinical application of these achievements.

### Anti-tumor effects of IL-6

Based on its pro-tumor effects, many primarily developed pharmaceuticals with completely IL-6 blockade were tested and used. However, these drugs improved symptoms at the cost of unpredictable side effects, presaging a multifaceted correlation among IL-6, tumor and body immune system. In fact, it was not until recent decades that the function of IL-6 in restraining tumor growth with hyperthermia came to be noticed. Daniel *et a*l. found that by using CT26 colorectal tumor, after treated with systemic thermal therapy (STT), marked lower tumor volume and a larger number of TUNEL^+^ apoptotic cells were detected in tumor vicinity. In the meantime, statistical analysis showed higher vessel density and more gathering cytotoxic T cells accompanied with similar IL-6 level alterations, suggesting the existence of close relationship between them. Then, to further block the function of several mediates related to the trafficking of CD8^+^T cells respectively, the results clarified the kernel role E/P-selectin and ICAM-1 played in the transference. To elucidate the exact part IL-6 participated, researchers established IL-6^-/-^ mice and verified comparably decreased E/P-selectin and ICAM-1 in contrast to the WT group after STT treatment. Some studies have corroborated the positive relevance between the expression level of E/P-selectin as well as ICAM-1 and tumor metastasis. Together with the findings here we can draw the conclusion that IL-6 inhibits tumor growth by promoting the expression of E/P-selectin and ICAM-1 via JAK/STAT3 pathway and consequently facilitating T cells infiltration. Notably, this effect was also witnessed in co-culture with STT treated mice tissues 133. Albeit a great breakthrough it may be, much unknown questions limited its clinical application. With many anti-tumor drugs targeted IL-6 as well as its downstream receptors, it is reasonable to query that to what degree and in what condition that the effect IL-6 induced by the thermal treatment can win against the one it plays in the pro-tumor activities.

**Figure 3:**
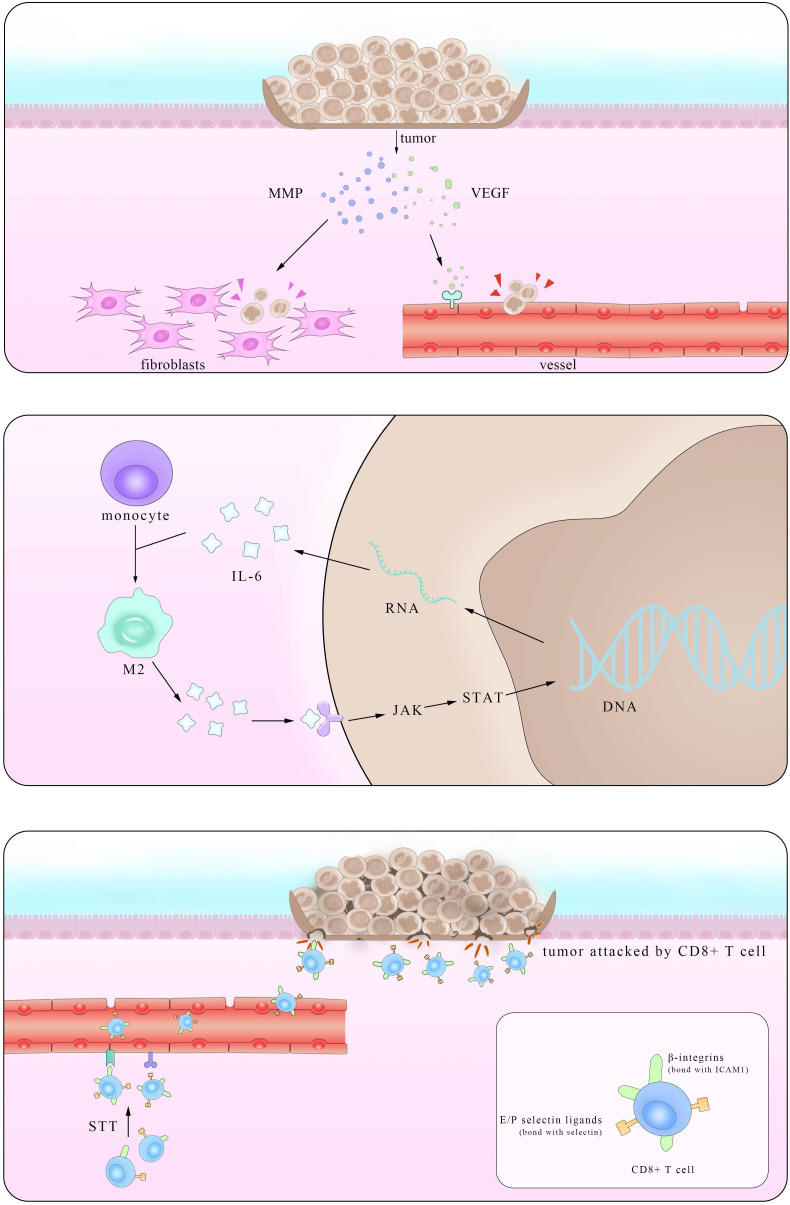
** Pattern of dual mechanism of pro-/anti-tumor IL-6**. The left part stands for the anti-tumor effect of IL-6 in promoting the secretion of VEGF and MMP in tumors and consequently enhancing their metastasis. The right part shows that after STT, higher expression levels of E/P selectin and ICAM1 facilitate T cells trafficking, thus restricting tumor progression.

In brief, the two main ways of IL-6 are not limited to intestine but also in various kinds of cancers, here, we just summarize the researches of IL-6 and its tumor-related functions specifically in the gut (as shown in Table 1). Also its dual functions are shown visually in Figure 3 below.

## Being associated with Parkinson's disease

Parkinson's disease (PD) is a common neurodegenerative disease occurred mostly in the aged. The main pathological manifestations witnessed in PD are the degeneration of dopaminergic (DA) neurons in the pars compacta of substantia nigra (SNpc) and marked reduction of DA level in corpus striatum. Similar to the close relationship between the nervous system and immune system in the intestine mentioned above, tremendous investigation have demonstrated that brain inflammation is one of the plausible explanations to the lesions of the dopaminergic neurons.

Based on the fact that some typical symptoms in PD like increasing colon transit time and constipation 147 resemble those in IBD, indicating the deficits in the intestinal immune barrier, and that some of them appear even earlier in the intestine than the motor ones finally manifest, researchers postulated the existence of brain-gut-axis modulation system in PD 148. Also concerning the direction by which the disease spreads, two views exist among scholars, which are from brain to the gut or vice versa 149.

The brain-gut-axis, an inevitable pathway for both directions of signaling, consists of both neural and humoral mediated pathways in acceptance to immune signals. The interactions between IL-6 and nerves have been discussed above in part 3.1 so here we put the emphasis other mediates included in the humoral pathway. With bacteria metabolites and other cytokines and cells able to cross the brain-blood-barrier (BBB) serving as neurotransmitters or neuromediators, the brain receives signals from the gut and send instructive projections back. As discussed before, IL-6 has close interactions with bacteria and play significant roles in the process of inflammation. However, without the ability to cross the BBB, the alteration of IL-6 concentration in the brain results largely from astrocytes and microglia in response to inflammation. And IL-6 in the intestine pass the inflammatory signal to the brain via other cells and molecules bearing the function of diffusing the inflammation, including CD4^+^T cells, Th17 cells and C-reactive protein (CRP), all of which form close link with IL-6 during inflammation 150. Of them the α-synuclein (α-SYN) may be the most direct one for its unique characteristics in nervous system, and it can go through the BBB from peripheral nervous system and blood in times of inflammation as well as in the opposite direction. Studies also found increase of α-SYN in both CNS and ENS in times of inflammation, but most experiments just focus on the relationship of the concentration of α-SYN monomers together with its toxic oligomers and IL-6 in the CNS, few researches we found on intestinal IL-6 include the one conducted by Gruden et al., which demonstrated a positive connection of serum level between them 151. In this way we deem IL-6 to be pro-inflammatory like loudspeakers aggravating the state of illness and setting up a vicious spiral. Another study found the adoption of a fibrillar form of α-SYN after treated with LPS and increasing IL-6 level correlated with TLR-4 152. Apart from the role in leading to apoptosis, IL-6 can sometimes be related to the plerosis of neurons and promote the neuronal survival after being treated 153. Yet the concrete connection between inflammation and α-SYN remains vague and whether IL-6 predominates in the process like that in CNS requires further investigation.

Although the concrete link between IL-6 and PD disease is yet to be found, we still hypothesize that IL-6 in the GI tract is strongly associated with PD through the modification of immunologic function and bacterial related factors. Careful analysis of these findings may provide a novel theraputic effect of the tricky problem.

## Conclusion

Owing to its various functions in almost the whole body, it is hard to restrict its level in the single intestine so the improvement here may be at the cost of defection elsewhere. Also on account of sophisticating environment of intestine, sorely alteration in IL-6 is likely to only alleviate the symptom and fundamental solution calls for a healthy diet. Despite the limitations, more clinical trials probably offer patients better choices with less side-effect.

In this review, we summarize the functions of IL-6 in intestinal immune barrier, which has extensive correlation with both nervous system and immune system. With its conflicting roles in inflammation and tumor, these will put forward possible direction for further investigation.

## Box 1: Key points

- Since its first clone and structural measurement in 1986, the functions of IL-6 in different organs have been gradually unveiled;
- The signaling pathway of IL-6 can be generally classified into three types, classic signaling, trans-signaling and trans-presentation;
- IL-6 contributes a lot to the maintenance of intestinal homeostasis by participating in the immune-epithelial-bacteria cross-talk and maintaining mucosal integrity;
- IL-6 has double roles in the process of intestinal inflammation and tumor, but the precise balance remains difficult to control;
- Unexpectedly exerting an influence on the peripheral nervous system, IL-6 within the intestine has potential effects on the Parkinson's disease.

## Figures and Tables

**Table 1:** Key factors in IL-6 related gastrointestinal tumor

Natural inhibitors	Inflammation initiators	Synthetical inhibitors
Ly6/Plaur domain-containing 8 (LYPD8) 134	Monocarboxylate transporter 4 (MCT4) 135	Tocilizumab (TCZ) 136
6,7-dimethoxy-1,2,3,4-tetrahydro-isoquinoline-3-carboxylic acid (M1) 137	Glucagon-like peptide-1 (GLP-1) 138 Glucose‑dependent insulinotropic polypeptide (GIP) incretins 138	Silibinin 139 [herbal]
	Corticotrophin-releasing hormone (CRH)/CRHR1 140	KIOM-MA/MA128 141 [herbal]
	Toll‐like receptor 4 142	Cocoa 143
	Sonic hedgehog (SHH) signaling 144	IL-6 monoclonal antibody (mAb) 145
		Probiotic agent VSL#3 146; Balsalazide (BSZ) 146

## Abbreviations

GI: Gastrointestinal
BSF-2: B-cell stimulatory factor 2
NBRF: National Biomedical Research Foundation Database
Genetic Sequence Data Bank: GSDB
IL-6: Interleukin-6
LAB: lactobacillus
IgA: immunoglobulin A
SCFAs: short chain fatty acids
MUC2: mucin2
TLRs: toll-like receptors
TJ: tight junction
JAM: junctional adhesion molecule
ZO: zonula occludens
JNK: c-Jun N-terminal kinase
Cdx2: caudal-related homeobox-2
CD: Crohn disease
UC: ulcerative colitis
HS/R: hemorrhagic shock and resuscitation
WT: wild type
KO: knock out
sPLA2: secretory phospholipase A2
PL: phospholipid
PE: phosphatidylethanolamine
SM: sphingomyelin
PC: phosphatidylcholine
LPC: lysophosphatidylcholine
Tregs: T regular cells
IL-6R: IL-6 receptor
gp130: glycoprotein130
sIL-6R: soluble IL-6R
STAT3: signal transducer and activator of transcription
SHP-2: Src homology 2 domain-containing protein tyrosine phosphatase-2
ERK: extracellular-signal-regulated kinase
MAPK: mitogen-activated protein kinase
PI3K: phosphoinositol-3 kinase
PkB: protein kinase B
SOCS 3: suppressor of cytokine signaling
PIAS: protein inhibitors of activated STAT
TGF-β: transforming growth factor β
ERK: extracellular signal-regulated kinases
VIP: vasoactive intestinal peptide
CRF: corticotropin-releasing factor
IBS: irritable bowel syndrome
uro2: urocortin 2
LPS: Lipopolysaccharide
HPA: hypothalamic pituitary adrenal axis
LMMP: longitudinal muscle myenteric plexus
RORγt: retinoid-related orphan receptor γt
CDAI: Crohn's Disease Activity Index
ICAM1: intercellular cell adhesion molecule-1
EPCR: endothelial protein C receptor
TM: thrombomodulin
VEC: vascular endothelial cell
Mφ: macrophages
CCL-20: CC-chemokine-ligand-20
CAC: colitis-associated colorectal cancer
CCR-6: CC-chemokine-receptor-6
tRXRα: truncated Retinoid X receptor-α
ADAM17: a disintegrin metalloprotease 17
VEGF: vascular endothelial growth factor
MMP: matrix metalloproteinase
STT: systemic thermal therapy
